# Relationships among cyberbullying, parental attitudes, self-harm and suicidal behavior among adolescents: results from a school-based survey in Vietnam

**DOI:** 10.1186/s12889-020-08500-3

**Published:** 2020-04-10

**Authors:** Hoang Thuy Linh Nguyen, Keiko Nakamura, Kaoruko Seino, Van Thang Vo

**Affiliations:** 1 Department of Global Health Entrepreneurship, Division of Public Health, Graduate School of Medical and Dental Sciences, Tokyo Medical and Dental University, 1-5-45 Yushima, Bunkyo-ku, Tokyo, 113-8519 Japan; 2 Faculty of Public Health, Hue University of Medicine and Pharmacy, Hue University, Hue, Vietnam; 3 Institute for Community Health Research, Hue University of Medicine and Pharmacy, Hue, Vietnam

**Keywords:** Parental attitude, Self-harm, Suicidal behaviors, Cyberbullying, Adolescents, Vietnam

## Abstract

**Background:**

The rapid and widespread development of social networking sites has created a venue for an increase in cyberbullying among adolescents. Protective mechanisms and actions must be considered, such as how proximal family factors can prevent self-harm and suicidal behaviors among adolescents exposed to cyberbullying. The present study examined the associations among cyberbullying, parental attitudes, self-harm, and suicidal behaviors after adjusting for confounding factors.

**Methods:**

Data were obtained from a school-based survey of randomly selected grade 6 students (11 years old) performed in Hue City, Vietnam, in 2018. A total of 648 students were interviewed face-to-face using a structured questionnaire based on the Global School-based Student Health Survey (GSHS). Univariate, multivariable logistic regression analyses were performed at 95% confidence level.

**Results:**

After adjusting for gender, perceived academic pressure, unhealthy behaviors, use of Internet devices, school bullying, and family living situation, a significantly higher risk of self-harm was detected among those who had experienced cyberbullying (adjusted odd ratio [AOR] = 2.97; 95% CI, 1.32–6.71). Parental acceptance retained a significant association with self-harm and suicidal behavior (*P* < 0.05) while parental concentration did not exhibit a significant association in a multivariable logistic regression model. In addition, suicidal ideation and suicidal planning were associated with an interaction effect between cyberbullying and parental concentration (AOR = 0.37; 95% CI, 0.15–0.94 and AOR = 0.23; 95% CI, 0.06–0.87, respectively).

**Conclusion:**

Cyberbullying has become an important phenomenon associated with self-harm among young adolescents in developing countries, and parental acceptance in proxy of parental attitude was positively related with severe mental health issues among adolescents. Thus, sufficient attention in efforts to promote adolescent health should be focused on family factors in the digital era of developing countries.

## Background

Self-harm and suicide are public health issues in young people [1], and tend to emerge during early adolescence, with rates of self-harm being high in the teenage years [2, 3] and suicide being the second most common cause of adolescent death after traffic accidents worldwide [4]. Hospital-reported cases account for only about one in eight adolescents who undergo self-harm in the community [2, 5]. In community-based studies, around 10% of adolescents reported a history of self-harm and some of these individuals reported some extent of suicidal intent underpinning their self-harm actions [5]. The lifetime prevalence of self-reported suicide attempts was 10.5% among European adolescents [6].

Self-harm and suicide in adolescents are the end-products of a complex process involving personality, social, and cultural factors [1]. Exposure to negative life events is a key factor associated with severe mental health [7]. Cyberbullying has become a widespread phenomenon in adolescence because of the rapid expansion of information and communications technology (ICT) [8], which can have an adverse impact on heath such as causing subsequent anxiety problems, self-harm, and suicide. Research on cyberbullying is still in its early stage, but the experience of being cyberbullied is known to cause a great deal of distress [9, 10]. Some previous studies have shown that adolescents who reported being the victim of bullying, or being involved in cyberbullying were more likely to engage in self-harm and suicidal behaviors [11, 12]. In addition, child and family adversity, maladaptive parenting, and parental divorce are negative factors associated with self-harm [11, 13]. Little is known about the importance of examining proximal family factors, particularly parental attitude, to gain further understanding of the possible pathways to self-harm and suicidal behavior. Hay and Meldrum (2010) have reported that the relationship between bullying victimization and non- suicidal self-injury (NSSI) was highly conditional, i.e., these associations disappeared almost completely in adolescents exposed to supportive parenting practices [14, 15].

Based on these earlier studies and rapid expansion of ICT, self-harm and suicidal behaviors among adolescents are considered to have associations with individual factors (gender, academic pressure, unhealthy behaviors, and internet device use), family factors (family living situation, parental attitude), and bullying (school bullying, cyberbullying).

Vietnam is a country in Southeast Asia that is undergoing rapid social-economical change and is becoming increasingly connected to the Internet, with younger people tending to use the Internet and commonly using social networking sites [16]. Although the family structure or role of parents has changed somewhat as a result of these social-economic changes in modern Vietnamese society [17], the impact of parent – adolescent relationships continues to plays a critical role in adolescent growth. However, there is a lack of evidence about the relationship between parental attitudes and severe mental problems among adolescents faced with dangers in a cyber environment.

Therefore, this study was performed to assess the associations among cyberbullying and self-harm and suicidal behaviors and to examine whether parental attitudes were associated with self-harm and suicidal behaviors among young adolescents exposed to cyberbullying.

## Methods

### Participants and data-collection

We used data from the baseline survey of the ongoing school-based cohort study being performed in Hue City (“Hue Healthy Adolescent Cohort Study” from 2018 to 2021). Participants were selected based on a multistage stratified cluster random sampling design. First, 5 junior high schools were randomly selected from a total of 23 public junior high schools in Hue City. Then, depending on the size of each school, 4–5 classes of students in the 6th grade (11 years old) were randomly chosen. A total of 755 students were invited to participate in the survey. The valid response rate was 86.83% (648 out of 755). Data were collected through face-to-face interviews using a structured questionnaire by the research team. To ensure consistent instruction and a consistent interview protocol, all the interviewees completed 1 day of health research training before the start of the study.

On the day of data collection, the study was again explained to the students. The students were reminded that the data being collected was anonymous and confidential and were told that they could stop the interview at any time. Students who had received parental consent and who had themselves consented to participate in the study were interviewed in the survey room. The interview took approximately 20–30 min to complete.

### Measures

The survey included a set of questions that had been developed by the Department of Global Health Entrepreneurship of Tokyo Medical and Dental University based on the Global School-based Student Health Survey (GSHS) [18]. The structured questionnaire elicited responses regarding the participant’s characteristics, daily activities, health risk behaviors, and other factors. The original English version of the questionnaire was translated into Vietnamese, which was then back-translated to English for confirmation.

Health-risk behaviors defined in this study included suicidal behaviors and self-harm. These behaviors among young adolescents were measured using the Young Risk Behavior Survey (YRBS) questionnaire developed by the Centers for Disease Control and Prevention (CDC) in the USA, which has been used in a number of studies of adolescents in Asian countries. The following were used to measure health-risk behaviors: (1) Suicidal Ideation was examined with the question “*In the past 12 months, have you ever seriously considered attempting suicide*?” (2) Suicidal Planning was examined with the question “*In the past 12 months, have you ever made a plan about how you would attempt suicide?*” (3) Suicide attempt was examined with the question “*In the past 12 months, did you actually attempt suicide?*” (4) Self-harm was identified with the question “*Have you ever deliberately hurt yourself in some way, such as cut or hit yourself on purpose or taken an overdose?”* All of these variables had a binary “yes” or “no” response.

A cyberbullying scale was used to assess the level of experience as a victim of cyberbullying in the previous 30 days. This scale for cyberbullying represents the respondent’s experience with six different forms of online bullying, with a combination of selected items in the original scale developed by Patchin and Hinduja in the USA as well as some new items [19]. The following items were included in this survey: (1) being called mean names/teased in a hurtful way; (2) being sent rude messages/pictures; (3) being left out/ignored by a group of friend; (4) having lies or rumors spread about you; (5) being put online the messages/photos/video about you; and (6) being threatened through communication technologies (cellphone, computers, email, and the Internet.). The possible responses to these questions were “never,” “once or twice,” “a few times,” “many times,” or “every day.” The final response regarding cyber-bullying was recorded as a “yes” for an answer of at least once for any experience of cyberbullying or “no” for an answer indicating no experience.

School bullying was defined as aggressive behavior by a student or group of students with a power imbalance and the potential to be repeated [18, 20]. (1) Having been bullied was identified with the question “*How many days were you bullied during the past 30 days*?” and the response was recorded as “yes” for an answer of one or more days or “no” for an answer indicating no experience.

The analysis also included a number of independent variables that may influence the likelihood of health-risk behaviors among adolescents: gender (male, female), family living situation, and use of Internet devices (< 1 h/day, 1–2 h/day, or > 2 h/day). The perceived level of academic pressure was measured with the question “*On average, how much academic pressure have you felt in the past 12 months?”* and a 5-point scale for possible responses ranging from 1 (almost none) to 5 (very much). The presence of unhealthy behaviors was determined as “yes” if a history of smoking, drinking alcohol or drug use was reported.

Perceived parental attitude was examined by the application of a principal component analysis (Varimax rotation) to six questions related to parents/guardians in the GHSH questionnaire, resulting in two dimensions. The reliability coefficient for each factor was less than 0.7, which is not uncommon for short scales of less than 10 items. According to Roe (1957) regarding the basic concept of parental attitude, “parental acceptance” and “parental concentration” were appropriate terms for naming the two dimensions of perceived parental attitude in this study [21]. Acceptance means that the parent regards the child as a full-fledged member of the family, neither concentrated upon nor overlooked, and that they encourage their child to fulfill his or her potential as best as possible. Concentration refers to the attitudes of parents who overprotect their children through restrictions upon their efforts to explore their environment and to meet others or who place heavy demands on their children to perform beyond their capacities and to achieve ambitious goals [22].

### Data analysis

In the descriptive analysis, categorical variables were summarized using proportions and were presented in tables, along with the significance of differences determined using the Pearson’s Chi square test.

The associations between self-harm/suicidal behavior and risk factors, including sociodemographic factors, bullying (school bullying, cyberbullying), and perceived parental attitude, were evaluated by calculating the crude odds ratio (OR) and the 95% confidence interval (CI) using univariate logistic regression analyses.

A multivariate logistic regression model was constructed to assess the association of independent variables with the likelihood that participants would report self-harm and suicidal behavior after adjusting for other variables (gender, perceived academic pressure, unhealthy behaviors, use of Internet devices, and family living situation). The Hormer and Lemeshow Goodness-of-Fit test with *P* > 0.05 was used to assess the goodness of fit model. The variance inflation factor (VIF) also showed no multicollinearity among independent variables. The data were analyzed using SPSS version 23.0 (SPSS Inc., Chicago, IL). In all the analyses, *P* < 0.05 was regarded as indicating statistical significance.

## Results

Table 1 shows the characteristics of 648 students divided according to their cyberbullying situation (52.3% male and 47.7% female). Nearly one tenth (9.0%) of the participants reported having been cyberbullied, while 17.6% reported having been the victim of school bullying. The majority of the respondents reported using of Internet devices for less than 1 h per day (57.3%). There were no significant differences in gender distribution, perceived academic pressure, family living situation, or use of Internet devices (*P* > 0.05) according to their cyberbullying situation. However, being a victim of school bullying and unhealthy behaviors differed significantly between respondents with and those without any experience of cyberbullying (*P* < 0.05).

**Table 1:** Descriptive statistics for cyberbullying situation among adolescents

		Total	Cyberbullying				***P***
			Yes 58 (9.0%)		No 590 (91.0%)		
		n (%)	n	%	n	%	
Gender	Male	339 (52.3)	26	7.7	313	92.3	0.232
	Female	309 (47.7)	32	10.4	277	89.6	
Perceived academic pressure	A little/None	432 (66.7)	36	8.3	396	91.7	0.564
	Some	123 (19.0)	11	8.9	112	91.1	
	Much	93 (14.4)	11	11.8	82	88.2	
Unhealthy behaviors	No	626 (96.6)	51	8.1	575	91.9	0.002^a^
	Yes	22 (3.4)	7	31.8	15	68.2	
Use of Internet devices (per day)	< 1 h	371 (57.3)	31	8.4	340	91.6	0.631
	1–2 h	232 (35.8)	23	9.9	209	90.1	
	> 2 h	45 (6.9)	4	8.9	41	91.1	
School bullying	No	534 (82.4)	37	6.9	497	93.1	0.000
	Yes	114 (17.6)	21	18.4	93	81.6	
Family living situation	Parents	567 (88.7)	52	9.2	515	90.8	0.805
	Mother or father	48 (7.4)	4	8.3	44	91.7	
	Others	33 (5.1)	2	6.1	31	93.9	
Self-harm	Yes	46 (7.1)	11	23.9	35	76.1	0.001^a^
	No	602 (92.9)	47	7.8	555	92.2	
Suicidal ideation	Yes	46 (7.1)	9	19.6	37	80.4	0.015^a^
	No	602 (92.9)	49	8.1	553	91.9	
Suicidal planning	Yes	19 (2.9)	4	21.1	15	78.9	0.081^a^
	No	629 (97.1)	54	8.6	575	91.4	
Suicide attempts	Yes	9 (1.4)	1	11.1	8	88.9	0.572^a^
	No	639 (98.6)	57	8.9	582	91.1	

^a^ Fisher’s Exact Test

Table 2 shows the results of an analysis with fitting to a logistic regression model for self-harm, suicidal behavior (suicidal ideation, suicidal planning, and suicide attempts) and cyberbullying. Among 7.1% of respondents who reported having experienced self-harm, the family living situation, perceived academic pressure, and history of unhealthy behavior, and cyberbullying were significantly associated with self-harm (*P* < 0.05) in a binary model. The rates of suicidal ideation and suicide attempts were significantly higher among adolescents with high levels of perceived academic pressure compared with those with no or little perceived academic pressure (OR = 3.15; 95%CI, 1.55, 6.39; OR = 8.13; 95%CI, 1.91, 34.62, respectively). Cyberbullying remained a significant predictor of self-harm with the addition of potential confounding factors to the multivariate model, such as gender, perceived academic pressure, unhealthy behaviors, use of Internet devices, and family living situation (adjusted odd ratio [AOR] = 2.97; 95%CI, 1.32, 6.71). In addition, a history of self-harm was significantly associated with suicidal behaviors after controlling for confounding factors (*P* < 0.05).

**Table 2:** Associations among cyberbullying, self-harm and suicidal behaviors among adolescents

	Self-harm		Suicidal Ideation		Suicidal Planning		Suicide Attempts	
	OR (95%CI)	AOR (95%CI)	OR (95%CI)	AOR (95%CI)	OR (95%CI)	AOR (95%CI)	OR (95%CI)	AOR (95%CI)
Gender								
Male	1.00	1.00	1.00	1.00	1.00	1.00	1.00	1.00
Female	0.92 (0.50, 1.67)	1.03 (0.54, 1.56)	2.17 (1.16, 4.07)^*^	2.30 (1.19, 4.44)^*^	1.53 (0.61, 3.85)	1.84 (0.68, 4.94)	2.22 (0.55, 8.95)	2.71 (0.59, 12.4)
Perceived academic pressure								
A little/None	1.00	1.00	1.00	1.00	1.00	1.00	1.00	1.00
Some	0.77 (0.31, 1.91)	0.75 (0.29, 1.94)	1.40 (0.63, 3.12)	1.41 (0.63, 3.19)	0.63 (0.14, 2.89)	0.66 (0.14, 3.06)	1.17 (0.12, 11.37)	1.25 (0.13, 12.53)
Much	2.44 (1.21, 4.93)^*^	2.22 (1.04, 4.74)^*^	3.15 (1.55, 6.39)^*^	3.12 (1.51, 6.69)^*^	2.64 (0.95, 7.33)	2.33 (0.79, 6.83)	8.13 (1.91, 34.62)^**^	7.76 (1.59, 37.86)^**^
Unhealthy behaviors								
No	1.00	1.00	1.00	1.00	1.00	1.00	1.00	1.00
Yes	7.02 (2.71, 18.23)^**^	6.39 (2.19, 18.61)^**^	3.09 (1.00, 9.54)^*^	2.99 (0.88, 10.14)	6.02 (1.62, 22.42)^*^	7.48 (1.68, 33.40)^*^	3.68 (0.44, 30.77)	7.23 (0.65, 80.34)
Use of Internet devices (per day)								
< 1 h	1.00	1.00	1.00	1.00	1.00	1.00	1.00	1.00
1–2 h	0.85 (0.44, 1.62)	0.80 (0.40, 1.61)	1.27 (0.67, 2.41)	1.30 (0.67, 2.54)	0.48 (0.16, 1.50)	0.43 (0.13, 1.42)	0.53 (0.11, 2.64)	0.58 (0.11, 3.24)
> 2 h	0.88 (0.26, 3.00)	0.76 (0.20, 2.85)	1.89 (0.68, 5.25)	1.95 (0.66, 5.71)	1.28 (0.28, 5.87)	1.13 (0.23, 5.67)	1.38 (0.16, 11.75)	1.74 (0.17, 17.30)
Family living situation								
Both parents	1.00	1.00	1.00	1.00	1.00	1.00	1.00	1.00
Mother or father	1.82 (0.68, 4.90)	2.03 (0.71, 5.75)	1.23 (0.42, 3.60)	1.26 (0.41, 3.85)	0.73 (0.10, 5.65)	0.73 (0.09, 5.98)	1.99 (0.24, 16.87)	2.22 (0.23, 21.75)
Others	4.22 (1.71, 10.42)^*^	5.26 (2.04, 13.60)^**^	1.35 (3.95, 4.63)^*^	1.58 (0.44, 5.65)	2.22 (0.49, 10.10)	2.54 (0.54, 12.05)	6.03 (1.17, 31.12)^*^	8.41 (1.38, 51.45)^*^
School bullying								
No	1.00	1.00	1.00	1.00	1.00	1.00	1.00	1.00
Yes	1.73 (0.87, 3.46)	1.46 (0.69, 3.08)	1.52 (0.75, 3.10)	1.17 (0.54, 2.52)	1.70 (0.60, 4.83)	1.46 (0.48, 4.40)	3.85 (1.02, 14.56)	3.05 (0.71, 13.04)
Cyberbullying								
No	1.00	1.00	1.00	1.00	1.00	1.00	1.00	1.00
Yes	3.71 (1.77, 7.78)^*^	2.97 (1.32, 6.71)^*^	2.75 (1.25,6.02)^*^	2.10 (0.90, 4.88)	2.84 (0.91, 8.86)	1.90 (0.56, 6.43)	1.28 (0.16, 10.39)	0.55 (0.06, 5.43)
Self-harm								
No	–	–	1.00	1.00	1.00	1.00	1.00	1.00
Yes	–	–	7.79 (3.79, 16.04)^*^	6.93 (3.09, 15.56)^*^	18.30 (7.00, 47.86)^*^	14.37 (4.95, 41.73)^*^	18.23 (4.72, 70.49)^*^	10.95 (2.27, 52.93)^*^

AOR adjusted odds radio (adjusted for gender, perceived academic pressure, unhealthy behaviors, use of Internet devices, family living situation, school bullying)

^*^P < 0.05; ^**^P < 0.001

As shown in Table 3, self-harm and suicidal behaviors showed significant associations with perceived parental attitude, including acceptance and concentration (*P* < 0.05). Moreover, parental acceptance retained a significant positive association with regard to reducing self-harm and suicidal behaviors, while parental concentration did not have a significant association in a multivariable logistic regression model (*P* < 0.05). Adolescents with more perceived parental acceptance were 0.52 times less likely to engage in self-harm (AOR = 0.52; 95% CI, 0.38–0.71), 0.35 times less likely to engage in suicidal ideation (AOR = 0.35; 95% CI, 0.25–0.48), 0.33 times less likely to engage in suicidal planning (AOR = 0.33; 95% CI, 0.20–0.53), and 0.25 times less likely to engage in suicidal attempts (AOR = 0.25; 95% CI, 0.11–0.58) compared with those with less perceived parental acceptance. In the interaction model, however, adolescents who had experienced cyberbullying and had more perceived parental concentration were 0.37 times less likely to engage in suicidal ideation (AOR = 0.37; 95% CI, 0.15–0.94) and 0.23 times less likely to engage in suicidal planning (AOR = 0.23; 95% CI, 0.06–0.87) than those with no experience of cyberbullying and less perceived parental concentration (Table 3).

**Table 3:** Associations among self-harm, suicidal behavior and perceived of parental attitudes after adjusting gender, perceived of academic pressure, school bullying, use of Internet devices, unhealthy behaviors, and family living situation

	Self-harm	Suicidal Ideation	Suicidal Planning	Suicide Attempts
**Crude model**	**OR (95%CI)**	**OR (95%CI)**	**OR (95%CI)**	**OR (95%CI)**
Factor 1_parental acceptance	0.50 (0.38, 0.67) ^*^	0.33 (0.24, 0.45) ^*^	0.31 (0.20, 0.48) ^*^	0.25 (0.13, 0.48) ^*^
Factor 2_parental concentration	1.11 (0.82, 1.50)	1.37 (1.01, 1.87)^*^	1.95 (1.21, 3.15) ^*^	1.86 (0.95, 3.67)
**Multivariate model**	**AOR (95%CI)**	**AOR (95%CI)**	**AOR (95%CI)**	**AOR (95%CI)**
Cyberbullying				
No	1.00	1.00	1.00	1.00
Yes	2.89 (1.24, 6.74)^**^	1.92 (0.76, 4.84)	1.47 (0.38, 5.68)	0.47 (0.04, 5.24)
Factor 1_parental acceptance	0.52 (0.38, 0.71)^*^	0.35 (0.25, 0.48) ^*^	0.33 (0.20, 0.53) ^*^	0.25 (0.11, 0.58) ^*^
Factor 2_parental concentration	0.99 (0.73, 1.34)	1.19 (0.87, 1.62)	1.62 (0.99, 2.68)	8.94 (1.81, 44.20)^**^
**Interaction model**	**AOR (95%CI)**	**AOR (95%CI)**	**AOR (95%CI)**	**AOR (95%CI)**
Cyberbullying				
No	1.00	1.00	1.00	
Yes	4.77 (1.97, 11.51)^**^	3.13 (1.05, 9.33)^*^	5.82 (1.15, 29.54)^*^	–
Factor 1_parental acceptance	0.44 (0. 30, 0.62)^**^	0.33 (0.23, 0.48)^**^	0.27 (0.15, 0.49)^**^	0.29 (0.12, 0.71)^*^
Factor 2_parental concentration	1.11 (0.79, 1.54)	1.36 (0.97, 1.90)	2.14 (1.19, 3.83)^*^	1.64 (0.75, 3.58)
Cyberbullying x Factor 1_parenal acceptance	1.93 (0.94, 3.98)	1.23 (0.54, 2.80)	1.86 (0.67, 5.17)	–
Cyberbullying x Factor 2_parental concentration	0.53 (0.23, 1.22)	0.37 (0.15, 0.94)^*^	0.23 (0.06, 0.87)^*^	–

AOR adjusted odds radio (adjusted for gender, perceived academic pressure, unhealthy behaviors, use of Internet devices, family living situation, school bullying)

^*^P < 0.05; ^**^P < 0.001

## Discussion

The results of the present study conducted in Vietnam showed that 9.0% of junior high school students reported their experience of being cyberbullied, and that experience of being cyberbullied was significantly associated with self-harm. In addition, parental acceptance showed a significant protective relationship by reducing the rates of self-harm and suicidal behaviors, including suicidal ideation, suicidal planning, and suicide attempts in a sample of adolescents from Hue city, Vietnam.

The influence of rapid advances in the Internet as well as mobile technologies is not limited to developed countries. Such advances are also occurring in developing countries, and there is increasing research interest in comparing bullying in school and online environments [23–25]. The percentages of reported experience of being victims of traditional school bullying and cyberbullying among adolescents in this study, 17.6 and 9.0%, were smaller than what were reported in other studies in the US, Vietnam, and Serbia, ranged from 20.1 to 44.7% and 16.2 to 28.9%, respectively [26–28]. The risk of being victim of traditional bulling surpassed the risk of being victim of cyberbullying, consistently [23, 26, 27, 29]. Some authors have suggested that traditional bullying and cyberbullying are distinct phenomena, while others have suggested that they are similar [30]. Vietnamese students have not been sufficiently educated about bullying and cyberbullying. Bullying behaviors and joking or teasing behaviors can be confused quite easily. Therefore, apart from traditional bullying, it is important to note that cyberbullying is occurring and must be addressed.

Both traditional bullying and cyberbullying have been shown to have negative impacts on adolescent development [25, 26, 31, 32]. This study demonstrated a significant association between cyberbullying and self-harm among adolescent after adjusting for potential confounders, but no significant relationship was observed with suicidal behavior (suicidal ideation, suicidal planning, and suicide attempts) in this population. Although not surprising, this observation extends the international need for further research by confirming this correlation in the context of developing countries [12, 26, 33, 34]. Both victims and perpetrators of cyberbullying were shown to be more likely to have suicidal thoughts and to attempt suicide than those who were not involved [19]. Although these correlations indicate the impact of cyberbullying, there is still debate regarding the causal relationships between cyberbullying and mental health problems [35]. While clear evidence of an association between cyberbullying and mental health problems was obtained in cross-sectional studies, longitudinal studies are more conservative in confirming these associations [36, 37].

Our findings indicated the importance of perceived parental attitudes with regard to the risks of self-harm and suicidal behaviors among adolescents in a multivariable model, which included cyberbullying, school bullying, and other potential risk factors. Students perceived parental acceptance was significantly associated with self-harm, suicidal ideation, suicidal planning, and suicide attempts in the multivariable model. Regarding traditional bullying and self-harm, several studies have indicated that the relationships of both bullying and victimization with self-harm and of depressive mood with self-harm were moderated by parental support [14, 38]. Modern trends emphasize adolescents’ competence and needs for independence, but parental support plays a critical role in leading children to the next level of social functioning and promoting their mental health [39]. Asian culture generally emphasizes respect for authority, and parents tend to use more commands and attempt to control their children’s attention more directly than parents in Western cultures [40]. The results of the present study in Vietnam clearly indicated the importance of family or parental attitudes (acceptance or concentration) in regulating self-harm and suicidal behavior among adolescents; specifically, a high degree of understanding or acceptance from parents was associated with a reduced likelihood of mental issues, which was consistent with previous research [41, 42]. However, evidence regarding the moderating role of both dimensions of parental attitude proxies on the behavior of adolescents directly faced with cyberbullying remains insufficient. The present study only revealed that more parental concentration among adolescents who had experienced cyberbullying was associated with a lower likelihood of suicidal ideation and planning, compared with those with no experience. Therefore, when developing interventions related to bullying and poor mental health consequences, it is critical to include cyberbullying in this present era of rapid technology changes, and it may also be beneficial to include family-related factors.

The presently reported findings should be viewed in the context of the study’s limitations. First, suicidal behavior and self-harm are difficult to determine based only on interviews, particularly in the context of Vietnamese culture, and it is not sufficient to assess these situations through only the presence/absence of suicidal behavior and self-harm in adolescents over the previous 12 months. Therefore, future studies should also include other characteristics of self-harm and suicidal behavior (e.g., frequency or current practice). The second limitation was related to the use of an adolescent self-report questionnaire, and the internal consistency of several measures, such as cyberbullying and perceived parental attitudes, was relatively low (< 0.7). Third, the cross-sectional nature of the study means that it is not possible to establish causality. Consequently, the directions of the associations between parental attitudes and mental issues can be interpreted in both ways; increasing parental concentration happens in children having the severe mental health. In addition, our study excluded out-of-school adolescents, and adolescents studying in the private schools, thereby limiting the generalizability of the findings to all adolescents in Vietnam. Finally, the prevalence of suicide attempts was small in our study population, which might have influenced the associations. Further studies with larger sample sizes are required.

## Conclusions

Despite the limitations outlined above, the present study provided insight into the relationship between cyberbullying and self-harm, which has not attracted sufficient attention in efforts to promote health among adolescents compared with other topics. The present findings also indicated the important impact of parental attitude on mental health among young adolescents. An urgent need exists for evidence-based and compassionate programs to reduce bullying and thereby promote wellbeing among young people. The prevention of bullying should start in early childhood, and preventative measures should address its presence in online environments. The present findings should inform future longitudinal investigations of the roles of parents in protecting adolescents faced with various types of bullying, including cyberbullying.

## Data Availability

The generated dataset is available upon request to the corresponding author at the contact address in this article.

## Abbreviations

CDC: Centers for disease control and prevention
DSM-5: Diagnostic and statistical manual of mental Disorders
ICT: Information and communication technology
GSHS: Global school-based student health survey
SAVY: Survey assessment of Vietnamese Y outh
WHO: World Health Organization
